# Susceptibility of Leaves from Commercially Important Ornamental Shrubs to Artificial Inoculation with *Phytophthora ramorum*

**DOI:** 10.3390/life16060996

**Published:** 2026-06-12

**Authors:** Marco Fiaschetti, Alessandra Benigno, Beatrice Ginetti, Viola Papini, Salvatore Moricca

**Affiliations:** Department of Agricultural, Food, Environmental and Forestry Science and Technology (DAGRI), Plant Pathology and Entomology Section, University of Florence, 50144 Florence, Italy; marcofiaschetti@virgilio.it (M.F.); beatrice.ginetti@gmail.com (B.G.); viola.papini@unifi.it (V.P.); salvatore.moricca@unifi.it (S.M.)

**Keywords:** invasive oomycete pathogen, nursery plant trade, host susceptibility assessment, sporulating hosts, airborne inoculum, transmission risk, pathogen ecology, disease control

## Abstract

The quarantine pathogen *Phytophthora ramorum* has a high potential for dispersal due to its airborne inoculum, its wide range of hosts, and its ability to spread through the trade of nursery plants. For these reasons, it represents a serious threat to ornamental nursery production and, consequently, to urban, natural and semi-natural ecosystems. This oomycete pathogen (EU1 lineage, A1 mating type) has been detected on *Viburnum tinus* in a commercial nursery located in the Pistoia nursery district (PND) (Tuscany, central Italy), one of the main nursery areas for the production of ornamentals in Europe. Artificial inoculations were carried out in the laboratory under controlled conditions, following a standard detached-leaf assay protocol, on leaves of 16 ornamental shrub species commonly marketed by the PND. Disease severity was assessed, and susceptibility categories (high, moderate, low, and non-susceptible) were defined based on data collected at 7 and 14 days post-inoculation and validated through statistical analysis. Inoculated species exhibited variable levels of disease severity. The results confirmed the pathogen’s high virulence on *Viburnum tinus* and *Rhododendron* hybrid ‘Madame Masson’. The following species were also found to be highly susceptible: *Ilex aquifolium*, *Loropetalum chinense*, *Magnolia stellata*, *Osmanthus fragrans*, and *Trachelospermum jasminoides*. *Camellia japonica*, *Nerium oleander*, *Osmanthus heterophyllus*, *Prunus laurocerasus*, and *Rhododendron obtusum* showed moderate susceptibility. *Arbutus unedo*, *Laurus nobilis*, *Photinia fraseri* and *Syringa vulgaris* exhibited low susceptibility. At the end of the trial, no infected species fell into the non-susceptible categories. The oomycete proved particularly aggressive on *Ilex aquifolium*, the most susceptible host among those tested. This high susceptibility is a new finding that could have significant epidemiological implications. Our findings emphasize the need for rigorous phytosanitary surveillance in nursery systems, based on constant monitoring and the adoption of high-throughput diagnostic protocols, in order to implement effective and rapid control measures.

## 1. Introduction

The genus *Phytophthora* (Oomycetes, Stramenopiles) comprises a large and diverse group of soilborne and airborne microorganisms that rank among the most destructive plant pathogens worldwide. Species within this genus are responsible for root rot, bleeding stem cankers, shoot and branch dieback, general declines, and sudden death syndrome. These pathogens affect agricultural, forest and horticultural ecosystems, with severe ecological and economic consequences [1,2,3,4]. Their ecological success is largely attributed to flexible life cycles, broad host ranges, and an exceptional capacity to exploit humid environments. Many *Phytophthora* species produce long-lived survival structures such as oospores, chlamydospores, and mycelial aggregates, as well as motile zoospores that enable rapid dissemination and persistence in soil, water and plant debris [1,5].

Among these pathogens, *Phytophthora ramorum* stands out as one of the most aggressive and epidemiologically significant generalist species. Since its emergence in the 1990s, *P. ramorum* has caused widespread mortality of oaks and tanoaks in western North America, known as Sudden Oak Death (SOD), as well as devastating outbreaks in Japanese larch plantations in the United Kingdom, where it is referred to as Sudden Larch Death (SLD). The pathogen has also induced severe foliar and shoot blight, known as “ramorum leaf blight” and “ramorum dieback” in a wide range of ornamental plants in Europe and North America. Forest understorey plants and ornamentals play a major role in the ecology and spread of the pathogen [6,7,8,9].

Phylogeographic and population genetic studies indicate that twelve clonal lineages of the pathogen have been characterised to date: the European lineages (EU1 and EU2), the North American lineages (NA1 and NA2), and the Asian lineages, including the Japanese groups (NP1, NP2, and NP3) and Vietnamese groups (IC1, IC2, IC3, IC4 and IC5) [6,8,10,11,12,13].

**Figure 1:**
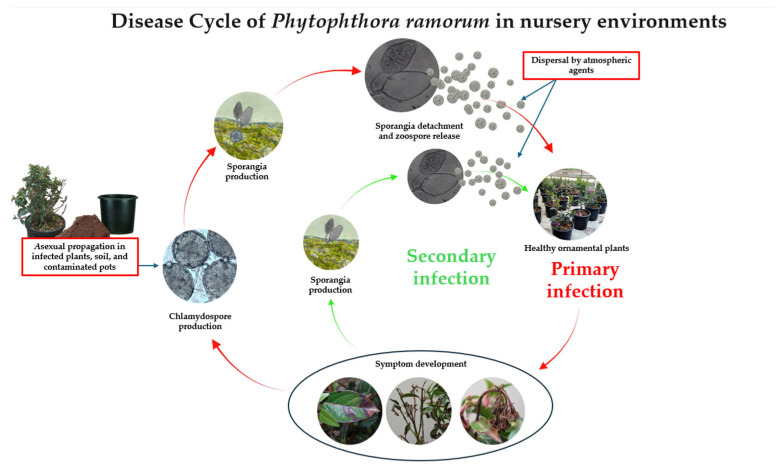
Disease cycle of *Phytophthora ramorum* in plant nursery production. The pathogen survives in infected plant tissue in the form of mycelium and chlamydospores. It also survives in leaf debris and soil as chlamydospores and sporangia. Under favourable conditions, sporangia release zoospores. These are dispersed by heavy rain, wind, and rain aerosols, thereby causing the primary infection of healthy plants. The development of symptoms, including petiole necrosis, shoot wilting and folding, stem browning, and cambium necrosis, is followed by new sporulation phases that sustain secondary infection cycles. The formation of chlamydospores (survival spores) is pivotal in ensuring the persistence of the pathogen in contaminated matrices and reactivating the infection chain in subsequent seasons or cultivation cycles. Red arrows indicate the primary infection cycle, green arrows indicate the secondary infection cycle.

The global spread of *P. ramorum* has been primarily driven by the international trade of infected ornamental plants, with nurseries acting as major hubs for long-distance dispersal and local microevolution [7,9,14]. Despite strict regulatory measures and eradication efforts, including quarantine, destruction of infected stock, sanitation and plant movement restrictions, *P. ramorum* continues to persist and re-emerge in nurseries and surrounding landscapes [15,16,17,18]. Latent infections, survival in potting media and leaf debris, and frequent reinfestations (secondary infection) limit the effectiveness of current management strategies (Figure 1).

At the landscape scale, eradication efforts in forests are logistically complex and economically demanding, and this explains why epidemics continue to expand in several regions [19,20,21,22].

The known host range of *P. ramorum* exceeds 170 plant species and continues to expand, with numerous woody ornamentals—particularly members of the Ericaceae—playing a central epidemiological role in nurseries [15,23,24,25]. Experimental inoculation studies have demonstrated pronounced interspecific variation in infection efficiency, lesion development and chlamydospore production among ornamental shrubs [26,27,28,29]. Some hosts function as highly efficient foliar sporulating species with limited mortality, acting as epidemiological amplifiers, whereas others develop lethal cankers but contribute less to pathogen spread [28,30]. However, for many widely traded ornamentals, quantitative data on foliar susceptibility, lesion development and the production of pathogen inoculum remain scarce. This lack of standardized susceptibility metrics hampers accurate pest risk analysis, epidemiological modelling and evidence-based prioritization of high-risk hosts for regulation and management [16,28,31].

An outbreak of *P. ramorum* caused by an EU1 clonal lineage (A1 mating type) isolate occurred in 2013 in the Pistoia nursery district (PND) (Tuscany, central Italy) [32]. PND is one of Europe’s leading ornamental plant production centres, to the extent that Pistoia is considered the European capital of ornamental plant cultivation [33,34]. The occurrence of *P. ramorum* highlighted the vulnerability of intensive nursery systems to *Phytophthora* invasions and their potential role as sources of pathogen spread throughout public and private green spaces and natural ecosystems [35,36,37]. Given this high potential for spread via the ornamental plant trade, quantitative data on host susceptibility and inoculum production are essential for understanding the epidemiological risk posed by *P. ramorum* and effectively managing that risk. Experimental studies on nursery crops and ericaceous ornamentals have demonstrated pronounced interspecific and cultivar-level variation in lesion development, infection frequency and chlamydospore production following *P. ramorum* inoculation [26,27,28,29]. Several ornamental hosts, including *Rhododendron*, *Viburnum* and *Prunus laurocerasus*, are known to sustain severe foliar infection and high levels of sporulation, thereby acting as “superspreading hosts” within nurseries and at planting sites [15,24,26,27,29,38]. However, for many widely traded ornamental species, susceptibility and sporulation potential remain poorly quantified. This knowledge gap constrains epidemiological modelling, pest risk analysis, and the evidence-based identification and prioritization of high-risk hosts for targeted regulation and nursery biosecurity measures [28,29,36].

This study provides valuable information on the susceptibility of some of the most widely traded and long-distance-transported ornamental shrub species. More specifically, the susceptibility of leaves from the sixteen most commonly sold species in the Pistoia nursery district to inoculation with *P. ramorum* was assessed in vitro by analysing lesion development and the final outcome of the infection. By generating comparable quantitative data across host species in a detached-leaf assay under controlled laboratory conditions, this work aimed to improve our understanding of their susceptibility and host–pathogen interactions, which are key aspects for supporting comparative host screening and informing risk-based management and regulatory strategies in ornamental plant production and marketing systems.

## 2. Materials and Methods

### 2.1. Host Plants and Preparation of Plant Material

The ornamental shrub species selected for this study are those with the highest economic significance (higher business volume) in the PND. The selection process involved a structured, multi-step approach: (i) compiling documented *Phytophthora ramorum* hosts from the international literature and official host lists; (ii) filtering for ornamental shrub species commonly affected in nursery and landscape environments; and (iii) identifying taxa that are most widely cultivated and commercially relevant within the PND production system. Accordingly, 16 shrub species were selected: *Arbutus unedo* L., *Camellia japonica* L., *Ilex aquifolium* L., *Laurus nobilis* L., *Loropetalum chinense* (R. Br.) Oliv., *Magnolia stellata* (Siebold & Zucc.) Maxim., *Nerium oleander* L., *Osmanthus fragrans* Lour., *Osmanthus heterophyllus* (G. Don) P.S. Green, *Photinia* × *fraseri* Dress, *Prunus laurocerasus* L., *Rhododendron* hybrid ‘Madame Masson’, *Rhododendron obtusum* (Lindl.) Planch., *Syringa vulgaris* L., *Trachelospermum jasminoides* (Lindl.) Lem. and *Viburnum tinus* L. All shrub species included in this study are recognized or suspected hosts of *P. ramorum* and are commonly found in nurseries as well as in public and private urban green spaces. Plant material was collected during spring and summer from the G.E.A. Green Economy and Agriculture—Centro per la Ricerca s.r.l. (Pistoia, Italy) and from commercial nurseries within the PND. Leaves were collected from 10 plants of each species, for a total of 40 leaves per species (4 leaves per plant, taken from the 4 cardinal directions). Individual plants were considered independent replicates, while the leaves were treated as technical replicates.

Of these, 30 leaves received the inoculation treatment and 10 served as negative controls. Samples were transported to the laboratory immediately under cool conditions and processed within 24 h to preserve tissue integrity and minimize post-harvest tissue physiological impairment.

### 2.2. Pathogen Isolate and Inoculum Preparation

Twelve isolates of *P. ramorum* (EU1 clonal lineage, A1 mating type) were used for the initial screening to select the strain to be inoculated [32]. Following a preliminary screening focused on growth rate at 24 °C and pathogenicity on *V. tinus* leaves alone, isolate SB05a (GenBank accession KF181162) was selected for inoculation tests, as it exhibited the highest virulence, defined as the speed of colonisation of leaf tissue. This was done to ensure uniform and measurable infection of all host plants tested. The SB05a isolate selected for this study was recovered in 2013 from symptomatic *Viburnum tinus* plants at a commercial nursery in Pescia (Pistoia Province, Italy), where it caused a documented outbreak. The isolate was routinely subcultured onto V8 agar (V8A; 100 mL V8 vegetable juice, 900 mL deionized water, 3 g L^−1^ CaCO_3_, 16 g L^−1^ agar) and incubated at 20 °C in the dark, which are conditions suitable for optimal growth and viable inoculum production. A detailed description of the macroscopic and microscopic characteristics of this isolate has been provided. To evaluate colony morphology across different media, mono-hyphal isolates incubated for 7 days at 23 °C were transferred to V8A, malt extract agar (MEA, DIFCO, Detroit, MI, USA) and potato dextrose agar (PDA, DIFCO, Detroit, MI, USA). Sporangial production was stimulated by transferring square agar plugs (approximately 1 cm^2^) from 5–7-day-old colonies into sterile filtered pond water. Actively growing colonies were periodically transferred to fresh medium to maintain vigour and standardization for the artificial inoculation tests. For pathogenicity assays, twenty replicate cultures of the selected isolate were prepared on carrot agar (CA) medium, prepared using fresh carrots and agar, and incubated for 7–10 days at 22 °C in the dark. Mycelium agar plugs (5 mm in diameter) were aseptically excised from the edge of an actively growing colony on CA using a sterile cork borer, before being immediately applied to detached leaves in accordance with the established pathogenicity assay protocol. Sterile CA plugs of the same size, obtained from four uninoculated Petri dishes, were used to inoculate control leaves. Inoculated leaves were maintained in sealed humid chambers to ensure high relative humidity and incubated in a thermostatically controlled chamber at 22 °C in the dark for the duration of the experimental period.

### 2.3. Detached Leaf Inoculation Assay

For detached-leaf inoculation assays, inoculated samples were kept separately according to species, treatment and control group in sealed humid chambers to prevent cross-contamination. Artificial wounds were produced using a standardised protocol to ensure consistent infection conditions, with incisions of a consistent length and depth to enable reproducible and comparable results [39,40,41]. Leaves were collected from ten plants per species (biological replicates), with four leaves sampled from each plant (one from each cardinal direction), for a total of forty leaves per species. Each leaf (technical replicate) was placed individually in a sterile 92 × 16 mm Petri dish containing moistened sterile absorbent paper supplemented with 2 mL of sterile distilled water in order to maintain the near-saturated humidity conditions that are favourable for *P. ramorum* infection. Each Petri dish represented a single experimental unit. The dishes were then sealed and incubated in the dark at 22 °C for 14 days in a thermostatically controlled chamber.

### 2.4. Disease Assessment and Data Collection

Pathogenicity of *P. ramorum* was assessed on each tested species using detached leaf assays. Inoculated leaves were incubated under uniform high-humidity conditions, and disease development was assessed at 7 and 14 days post-inoculation (dpi) according to three parameters (Table 1): disease incidence, defined as the percentage of leaves exhibiting necrosis (parameter 1); disease severity, quantified by digital image analysis and expressed as percentage of necrotic area out of total leaf area (parameter 2); and lesion size, measured by determining the maximum length along the midrib and the maximum width perpendicular to the midrib and expressed as percentages (parameter 3) according to protocols commonly adopted in detached leaf assays for *Phytophthora ramorum* [26,40,41].

- Lesion length along midrib = $L_{lesion}$ (mm)

- Total leaf length (midrib) = $L_{leaf}$ (mm)

$$\%\;\mathrm{Lesion}\;\mathrm{length}=\left(\frac{L_{lesion}}{L_{leaf}}\right)\times 100$$

- Lesion width = $W_{lesion}$ (mm)

- Total leaf width = $W_{leaf}$(mm)

$$\%\;\mathrm{Lesion}\;\mathrm{width}=\left(\frac{W_{lesion}}{W_{leaf}}\right)\times 100$$

Asymptomatic leaves (with a lesion length equal to zero) were included in the analysis. The re-isolation of pathogens was conducted on four to six tissue fragments per leaf, with the objective of confirming the positivity of infection. This positivity was expressed as the percentage of successful recoveries. The recovered oomycete cultures were identified on the basis of micro-morphological traits, and this identification was subsequently confirmed by means of amplification and sequencing of the internal transcribed spacer (ITS) region. Control leaves were assessed in parallel.

### 2.5. Data Analysis

All statistical analyses were performed using SPSS Statistics 28 (IBM Corp., Endicott, NY, USA). Data were preliminarily tested for normality and homogeneity of variances using the Shapiro–Wilk and Levene’s tests, respectively. When necessary, percentage data were log-transformed to satisfy the assumptions required for parametric analyses. Differences in disease severity among isolates and plant species were analysed by one-way analysis of variance (ANOVA). Disease severity was assessed as a percentage of necrotic leaf area, lesion length, and lesion width at 7 and 14 days post-inoculation (dpi). Mean comparisons were performed using Tukey’s honest significant difference (HSD) post hoc test. Disease incidence, expressed as the proportion of symptomatic leaves, was analysed using the χ^2^ (chi-square) test. Results were considered statistically significant at *p* < 0.05. Data are presented as mean ± standard deviation (SD), and asymptomatic leaves were included in all analyses.

## 3. Results

### 3.1. Screening of Phytophthora Ramorum Isolates

**Figure 2:**
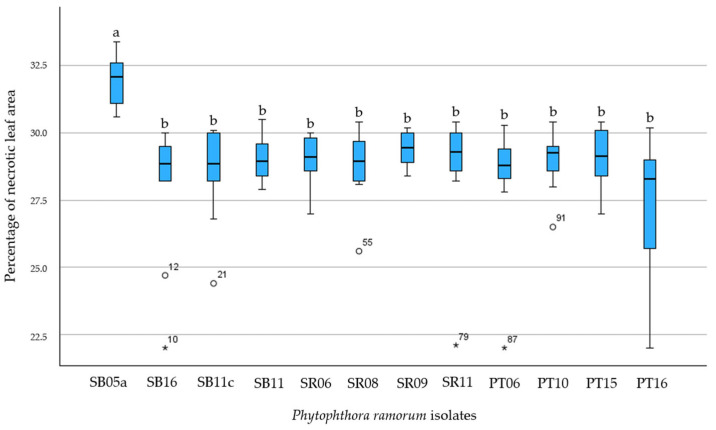
Boxplot showing the percentage of necrotic leaf area on *Viburnum tinus* leaves at 7 days post-inoculation for different *Phytophthora ramorum* isolates. Boxes represent the interquartile range (IQR), the central line indicates the median, whiskers show the range of non-outlier values, and points represent outliers. Different lowercase letters above the boxplots indicate significant differences among isolates according to the post hoc test (*p* < 0.05); isolates sharing the same letter are not significantly different. Numbers adjacent to outliers indicate observation (case) identifiers. Significant variability in aggressiveness among isolates is observed, with SB05a showing the highest median necrotic area.

Twelve *P. ramorum* isolates were initially screened to select the strain for subsequent inoculation assays. The preliminary evaluation was based on the percentage of necrotic leaf area at 7 days post-inoculation. Disease severity varied among isolates, with statistically significant differences detected (*p* < 0.05). Among those tested, isolate SB05a (GenBank accession KF181162) showed the highest aggressiveness, as indicated by consistently greater lesion development compared to the other isolates. This result was further supported by pathogenicity tests on *V. tinus* leaves, where SB05a exhibited faster colonisation and more extensive lesion expansion. Different letters in Figure 2 indicate statistically significant differences among isolates (*p* < 0.05) according to one-way ANOVA followed by Tukey’s post hoc test.

**Figure 3:**
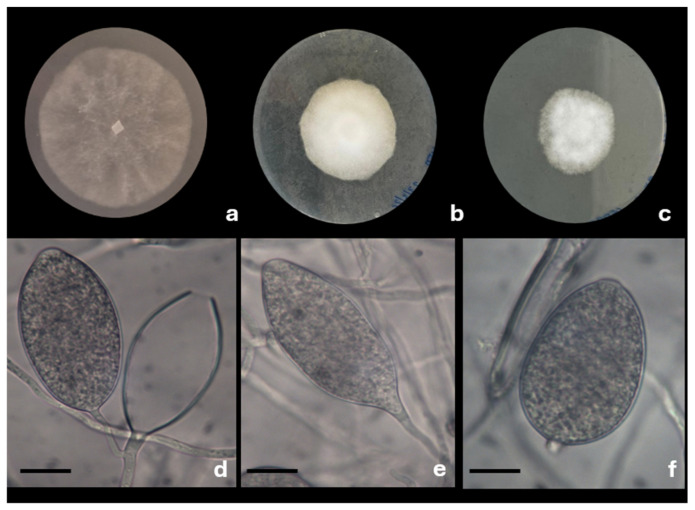
Mycelial growth and microscopic characteristics of *Phytophthora ramorum* (SB05a). (**a**–**c**) Colony morphology on different culture media (from left to right): V8A, PDA and MEA. Differences in colony appearance, texture and growth pattern and well as in mycelium compactness can be observed; (**d**–**f**) sporangia variable in shape: (**d**) semipapillate sporangia borne on a single sporangiophore after 24 h flooding in filtered pond water (scale bar = 25 μ); (**e**) elongated, semipapillate sporangium (scale bar = 25 μ); (**f**) semipapillate caducous sporangium with short pedicel (scale bar = 25 μ).

Based on these results, the SB05a isolate was selected for subsequent inoculation assays. Colony morphology varied according to the culture medium used. On V8A, colonies were slightly stellate with a moderately cottony aerial mycelium (see Figure 3a). The colonies had a more compact appearance and a cottony aerial mycelium and displayed relatively slow and uniform growth when grown on potato dextrose agar (PDA) (Figure 3b). On malt extract agar (MEA), the colonies grew even more slowly and had a less cottony texture (Figure 3c). Both terminally and intercalarily, abundant globose chlamydospores were produced on V8A and CA, with an average diameter of 54.7 ± 8.5 μm. Semipapillate, caducous sporangia developed within 24 h, occurring singly or occasionally in pairs on sporangiophores with rounded-to-conical bases. Sporangia measurements (*n* = 100) averaged at 56.2 ± 9.5 × 29.3 ± 4.3 μm, with a mean length-to-breadth ratio of 1.9 ± 0.3 and an average exit pore diameter of 7.0 ± 1.0 μm. Considerable variation in sporangial morphology was observed, including ellipsoidal, lemon-shaped, ovoid, obovoid and ampulliform forms (see Figure 3d–f).

### 3.2. Temporal Development of Disease in Detached-Leaf Assays

**Figure 4:**
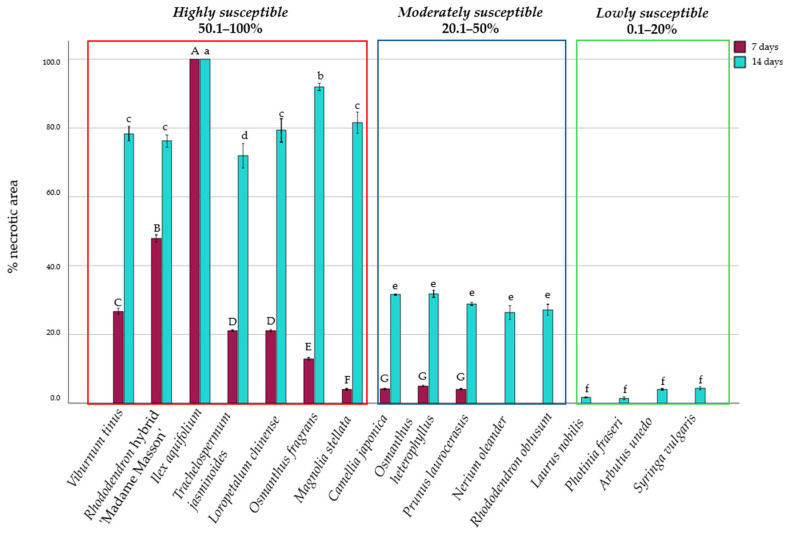
Percentage of necrotic area recorded on different plant species at 7 and 14 days post-inoculation. The species are grouped into three susceptibility classes: highly susceptible (red), moderately susceptible (blue), and lowly susceptible (green). Bars represent mean values ± standard error. Different letters indicate significant differences among species within the same evaluation time (*p* < 0.05).

Detached-leaf inoculation assays revealed significant differences in susceptibility to *P. ramorum* among the tested plant species (Figure 4). Disease severity, expressed as necrotic leaf area, differed significantly among species at both 7 and 14 days post-inoculation (dpi), as determined by one-way ANOVA (*p* < 0.05). For all susceptible species, disease severity increased over time, as indicated by both the proportion of symptomatic leaves and the expansion of necrotic lesions between 7 and 14 dpi, consistent with previous studies [26,27,28,29]. To facilitate comparison among species, susceptibility was categorized based on the percentage of necrotic leaf area at 14 dpi as follows: high susceptibility (50.1–100%), moderate susceptibility (20.1–50%), and low susceptibility (<20%). These thresholds were defined a priori to provide a biologically meaningful interpretation of disease severity and were consistent with the distribution of necrotic area observed across species (Figure 3). The classification was further supported by the statistical separation of species into distinct groups, as determined by Tukey’s HSD test (*p* < 0.05). In Figure 4, different letters indicate statistically significant differences among species at each time point according to Tukey’s HSD test (*p* < 0.05). At the end of the experiment, *P. ramorum* was successfully re-isolated from 99.1% of symptomatic leaves and confirmed by PCR, supporting its pathogenic role under the experimental conditions.

The rate of symptom development was assessed as the average lesion size over time (Table 2). Species differed not only in overall susceptibility but also in the timing and progression of lesion expansion. Among highly susceptible species, two distinct patterns were observed: some species (e.g., *Rhododendron* hybrid ‘Madame Masson’, *Ilex aquifolium*, and *Viburnum tinus*) exhibited rapid lesion development within the first 7 days followed by steady progression, whereas others (e.g., *Magnolia stellata* and *Trachelospermum jasminoides*) showed limited early development with a marked increase between 7 and 14 dpi. In moderately susceptible species, lesion expansion was generally slower and reached intermediate levels by 14 dpi, indicating partial restriction of pathogen spread. In contrast, low-susceptibility species displayed minimal lesion development, with lesions remaining small and localized throughout the experimental period.

### 3.3. Host Categorisation According to Susceptibility

**(A)**  **Species with high susceptibility**

**Figure 5:**
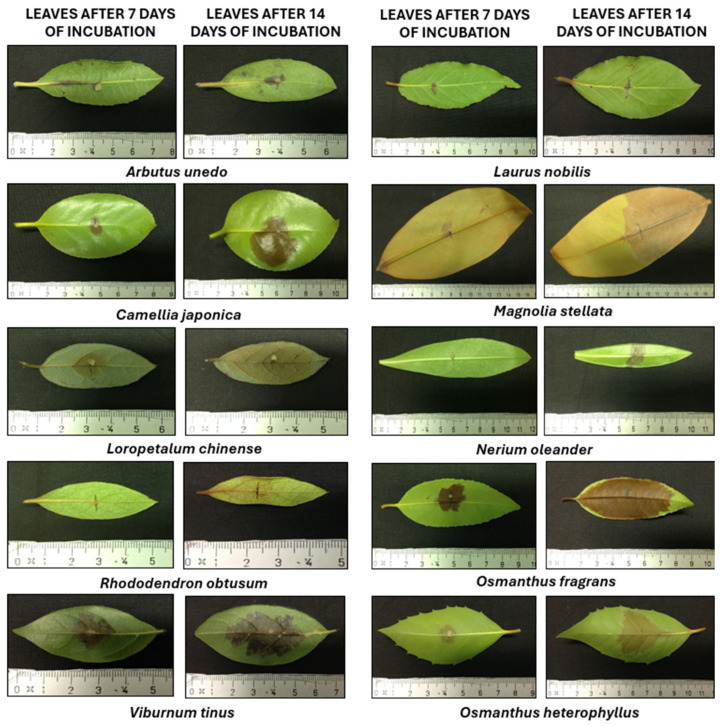
Disease symptoms, at 7 and 14 days of incubation, on detached leaves of the ornamental shrubs *Arbutus unedo*, *Laurus nobilis*, *Camellia japonica*, *Magnolia stellata*, *Loropetalum chinense*, *Nerium oleander*, *Rhododendron obtusum*, *Osmanthus fragrans*, *Viburnum tinus* and *Osmanthus heterophyllus* artificially inoculated with *Phytophthora ramorum* and incubated under controlled laboratory conditions. Differences in disease severity (necrotic leaf area) among the species over time are evident.

**Figure 6:**
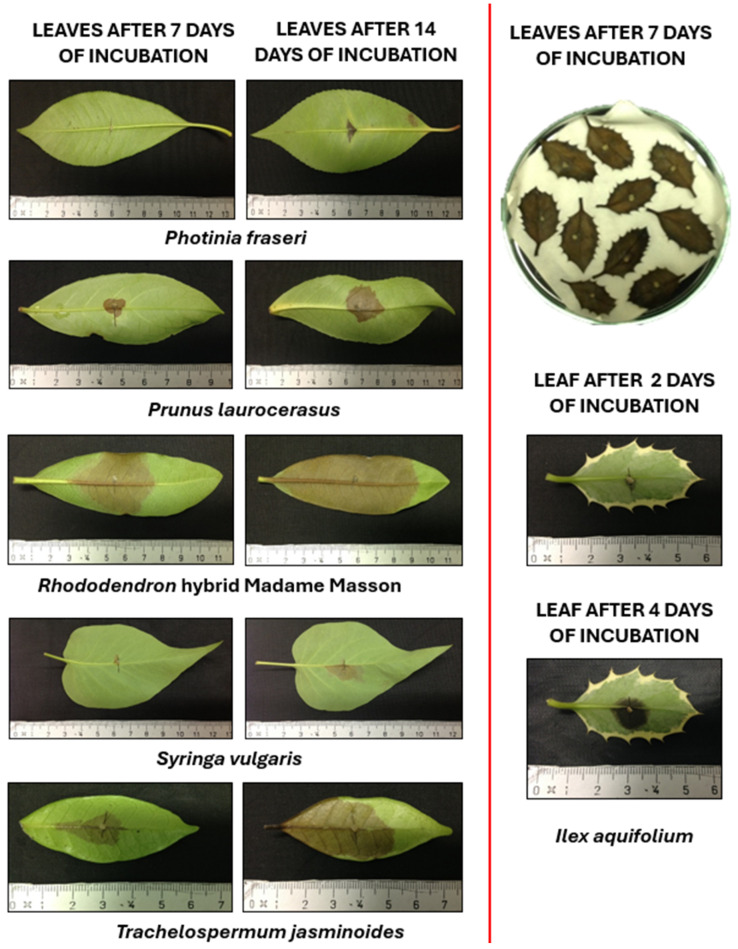
Images in the left-hand column: disease symptoms, at 7 and 14 days of incubation, on detached leaves of the ornamental shrubs *Photinia fraseri*, *Prunus laurocerasus*, *Rhododendron* hybrid ‘Madame Masson’, *Syringa vulgaris* and *Trachelospermum jasminoides* artificially inoculated with *Phytophthora ramorum* and incubated under controlled laboratory conditions. Images in the right-hand column: disease symptoms on a group of *Ilex aquifolium* leaves artificially inoculated with *P. ramorum* and incubated under controlled laboratory conditions in a 150 mm diameter glass Petri dish, at 7 days of incubation (top image). Detail of symptoms on individual leaves of *I. aquifolium* artificially inoculated with *P. ramorum* and incubated under controlled laboratory conditions, at 2 and 4 days of incubation (centre and bottom images, respectively). The temporal development of the lesions and of leaf necroses is a clear indication of the susceptibility of the species tested. The rapid development of symptoms on the leaves of *I. aquifolium* is apparent, with a very rapid progression of the infection already noticeable after 2 and 4 days of incubation.

*Rhododendron* hybrid ‘Madame Masson’, *Ilex aquifolium*, *Viburnum tinus*, *Magnolia stellata*, *Osmanthus fragrans*, *Loropetalum chinense* and *Trachelospermum jasminoides* leaves exhibited high susceptibility to *P. ramorum* (Figure 5). In these species, necrotic lesions developed rapidly following inoculation and expanded quickly over time. In many cases, lesions extended to cover the entire leaf lamina by 14 dpi (Table 2). Symptom development in *Ilex aquifolium* was particularly rapid, with all inoculated leaves showing necroses by 7 dpi. An additional time-course experiment revealed the appearance of visible lesions as early as 2 dpi, followed by rapid expansion by 4 dpi (Figure 6).

**(B)**  **Species with moderate susceptibility**

*Camellia japonica*, *Osmanthus heterophyllus*, *Prunus laurocerasus*, *Nerium oleander* and *Rhododendron obtusum* displayed intermediate levels of susceptibility. On the leaves of these species, lesion spread was slower, suggesting that infection was contained to some extent by host response. Initially, symptoms appeared as small, localised necrotic spots that, in some cases, continued to expand until they covered larger areas of the leaf blade. Complete leaf necrosis was observed in only some of the inoculated samples; in the others, disease development halted or remained confined to limited areas of tissue. Overall, these results suggest moderate susceptibility of these species.

**(C)**  **Species with low susceptibility**

*Arbutus unedo*, *Laurus nobilis*, *Syringa vulgaris* and *Photinia fraseri* exhibited low susceptibility, with delayed symptom expression. In leaves of these species, symptoms were either absent or restricted to small, localized necrotic areas even at 14 dpi (Figure 5 and Figure 6), indicating limited colonization of the leaf tissue by the pathogen. Where lesions were observed, they exhibited gradual development and did not substantially expand over the course of the study. The lesions remained restricted to the inoculation site or its immediate vicinity. In several samples, there was no coalescence of lesions or development to extensive leaf blade necrosis. Furthermore, there were no obvious signs of sporulation or widespread tissue collapse, confirming a limited and contained infection. These results indicate an effective host response, hindering the systemic spread of the pathogen and reducing its symptomatic impact.

## 4. Discussion

This study carried out a comparative assessment of the susceptibility of 16 ornamental shrub species to *P. ramorum* using detached-leaf assays under controlled laboratory conditions. By integrating symptom development, disease severity, and direct pathogen detection through re-isolation and molecular diagnostics, the research provided a reliable assessment of these hosts’ susceptibility and their potential epidemiological significance.

Pathogenicity tests on detached leaves are widely used to assess host susceptibility or resistance to pathogens. Multiple studies demonstrated that such an approach is effective in differentiating host genotypes (clones or varieties) on the basis of their resistance as well as to compare pathogen isolates for their virulence [42,43,44,45,46]. Lesion size and the extent of foliar necroses are parameters commonly used to quantify disease severity and pathogen aggressiveness. These metrics are frequently incorporated into indices that also account for the temporal dynamics of necrosis expansion [47,48,49,50,51]. These systems thus facilitate highly repeatable experimentation under controlled environmental conditions, a feature that sometimes provides more objective results than tests on whole plants. In whole-plant infection tests, in fact, the extent of necrotic tissue is influenced by host physiology, environmental conditions and inoculation methods, which may limit its reliability as an indicator of true disease severity in certain host–pathogen systems [40,44]. Consequently, several authors recommend interpreting foliar necrosis in conjunction with direct measures of pathogen colonization, such as detection of mycelium or pathogen DNA in infected tissues, assessment of sporulation capacity, or evaluation of infectivity in newly exposed tissues [47,51,52,53,54,55]. In this study, two sequential assessments conducted during the incubation period enabled a robust evaluation of species susceptibility and facilitated the reconstruction of symptom development curves for each host. This temporal approach enabled characterization of disease development dynamics across species, yielding valuable epidemiological insights [40]. Based on these analyses, the sixteen shrub species tested were classified into distinct susceptibility groups to *P. ramorum*. The inclusion of *Rhododendron catawbiense*, a species already known for its susceptibility, served as a practical reference for meaningful comparisons among infected host species.

Detached leaf assays remain a widely used and practical proxy for assessing relative host susceptibility. Lesion development rates have demonstrated reliability for evaluating *P. ramorum* virulence, with infection confirmed through pathogen re-isolation and molecular detection from symptomatic tissues, consistent with Koch’s postulates and standard diagnostic procedures for this regulated pathogen [56,57,58]. The assay thus provides a standardized and reproducible system where lesion size is a reliable indicator of host response, allowing comparisons among species. While sporulation or pathogen DNA may occasionally be detected in tissues with minimal necrosis, the overall ranking of susceptibility based on lesion development is consistent with previous studies [56,58,59]. Therefore, foliar necrosis should be regarded as a quantitative indicator of host damage, whereas pathogen re-isolation or PCR-based detection provides direct evidence of infective capacity. Integrating both metrics offers a reliable method for assessing host susceptibility and epidemiological risk in detached leaf assays [40,41,56,58]. The detached-leaf assays highlighted clear differences in susceptibility among the tested species. It is noteworthy that highly susceptible species developed extensive necrotic lesions, moderately susceptible species showed intermediate symptoms, and low-susceptibility species exhibited minimal or no visible symptoms. This apparent variability in host responses provides a quantitative framework for determining species susceptibility to *P. ramorum*, which could prove useful in future management strategies and risk assessments related to the marketing of nursery stocks.

After fourteen days of incubation, *Ilex aquifolium*, *Rhododendron* hybrid ‘Madame Masson’, *Viburnum tinus*, *Magnolia stellata*, *Osmanthus fragrans*, *Loropetalum chinense* and *Trachelospermum jasminoides* were classified as highly susceptible. These findings confirm the documented virulence of *P. ramorum* on *Viburnum* and *Rhododendron* species [60,61,62].

It is important to emphasise that *I. aquifolium* exhibited the highest susceptibility among the species tested, along with *T. jasminoides*. Our observations on *I. aquifolium* differ from those reported by Moralejo et al. [29,63], who reported this species to be only slightly susceptible, having observed limited lesion development and low variability. Discrepancies may be related to differences in experimental methodology and environmental conditions. We would tend to rule out any possible effect attributable to the pathogen lineage, not least because Moralejo et al. did not report any significant differences in virulence among the *P. ramorum* lineages tested. In their study, five isolates belonging to both the European EU1 and North American NA1 clonal lineages were used, whereas in the present study, only the EU1 isolate SB05a (A1 mating type) was employed, the only one currently reported from Italy [32]. Notably, Moralejo et al. [29] reported that the closely related species *I. canariensis* exhibits pronounced susceptibility, suggesting that susceptibility can vary considerably even among closely related species. In our study, *I. aquifolium* susceptibility was comparable to that of established high-risk hosts *Viburnum* and *Rhododendron*. Therefore, although *I. aquifolium* has not previously been reported as a natural host of *P. ramorum,* its widespread cultivation in nurseries and its widespread distribution as a component of the undergrowth flora of oak species in the Mediterranean environment suggest that this species should be closely monitored due to the risk that it could act as a driver of *P. ramorum* infections in Mediterranean oak forests if the oomycete were to escape human control. This is exactly what happened in North America: *P. ramorum* had been reported in areas surrounding nurseries before spreading epidemically throughout the natural environment of California and Oregon [61,62,63,64,65].

*T. jasminoides* also exhibited high susceptibility to artificial inoculation, corroborating previous detached leaf assays on evergreen ornamentals [26,66]. Its epidemiological significance is further supported by reports of natural infection in Oregon, where the pathogen has been detected in forest and nursery environments containing multiple *P. ramorum*-susceptible species [23,66,67].

The tested shrub species exhibited a broad spectrum of responses to infection, enabling their classification into four distinct susceptibility categories. Several taxa commonly identified as high-risk hosts, such as *Viburnum, Rhododendron* and *Camellia*, demonstrated high susceptibility, corroborating previous findings. In light of the global regulations governing *P. ramorum* and the pivotal role of the ornamental plant trade in its dissemination, these findings offer valuable insights for risk evaluation, surveillance prioritisation, and the development of more targeted and cost-effective phytosanitary strategies. Future research should focus on evaluating the sporulation potential of the pathogen and understanding how well a given host plant can acquire, support and transmit it (i.e., host competence). Such research is essential for confirming the in vitro trends observed and would be instrumental in developing more effective, targeted plant protection strategies. However, it should be noted that regulatory restrictions make in vivo testing on whole plants quite difficult since this is a quarantine pathogen.

## 5. Conclusions

Reports over the last decade in Tuscany of dangerous quarantined pests such as *Phytophthora ramorum*, *Xylella fastidiosa*, *Geosmithia morbida*, *Pityophthorus juglandis*, *Aleurocanthus spiniferus* and *Toumeyella parvicornis*, to name but a few, suggest that this region is particularly vulnerable to the introduction of invasive pests. Although it is always difficult to establish definite cause-and-effect relationships, the presence in Tuscany of one of the largest nursery districts in Europe suggests that it may play a role in this regard [68,69,70,71,72,73]. Studies on the susceptibility of commercially grown plants to the harmful oomycete *P. ramorum* in the PND must therefore be a priority. This study offers important insights into the susceptibility of the species selected for testing, which are among the most widely marketed in the public and private ornamental greenery sector. The findings provide a preliminary comparative assessment of host susceptibility under controlled experimental conditions and may help identify species deserving further investigation in more comprehensive studies. Furthermore, the identification of species capable of sustaining extensive foliar infection highlights potential differences in host suitability under the experimental conditions tested, which may serve as a basis for future studies aimed at evaluating pathogen spread and epidemiology under field conditions.

## Figures and Tables

**Table 1:** Definition of susceptibility categories on the basis of disease severity and lesion size parameters at the end of the experiment (14 days post-inoculation).

Susceptibility Categories	Disease Severity (% Necrotic Area)	Lesion Size (%)
Highly Susceptible	50, 1–100%	Large lesions (50, 1–100%)
Moderately Susceptible	20, 1–50%	Moderate lesions (20, 1–50%)
Lowly Susceptible	0, 1–20%	Small lesions (0, 1–20%)
Non-susceptible	0%	No lesions (0%)

**Table 2:** Mean lesion length (mm) at 7 (T1) and 14 (T2) days post-inoculation and corresponding relative increase (%) on leaves of the tested species and host susceptibility classification based on disease severity recorded at 14 days post-inoculation.

Species	T1 Average Lesion Size (mm)	T2 Average Lesion Size (mm)	Average Relative Increase (%)	Susceptibility Class
*Viburnum tinus*	26.87 ± 0.42	43.86 ± 0.32	63%	highly susceptible
*Rhododendron* hybrid ‘Madame Masson’	47.52 ± 1.02	76.92 ± 0.61	62%	highly susceptible
*Ilex aquifolium*	40.33 ± 0.32	40.33 ± 0.32	0%	highly susceptible
*Trachelospermum jasminoides*	18.02 ± 1.38	47.63 ± 0.63	164%	highly susceptible
*Loropetalum chinense*	11.06 ± 0.71	39.85 ± 0.64	260%	highly susceptible
*Osmanthus fragrans*	8.98 ± 1.05	31.85 ± 1.02	254%	highly susceptible
*Magnolia stellata*	3.99 ± 0.36	36.07 ± 0.75	804%	highly susceptible
*Camellia japonica*	3.76 ± 0.40	31.6 ± 0.63	740%	moderately susceptible
*Osmanthus heterophyllus*	5.01 ± 0.05	33.05 ± 0.38	560%	moderately susceptible
*Prunus laurocerasus*	4.02 ± 0.20	28.47 ± 0.18	608%	moderately susceptible
*Nerium oleander*	0	26.05 ± 0.33	-	moderately susceptible
*Rhododendron obtusum*	0	27.1 ± 0.69	-	moderately susceptible
*Laurus nobilis*	0	1.69 ± 1.01	-	lowly susceptible
*Photinia × fraseri*	0	1.37 ± 0.81	-	lowly susceptible
*Arbutus unedo*	0	3.97 ± 0.57	-	lowly susceptible
*Syringa vulgaris*	0	4.35 ± 1.13	-	lowly susceptible

## Data Availability

All data are included in the manuscript.
